# Functional Metabolomics Uncovers Metabolic Alterations Associated to Severe Oxidative Stress in MCF7 Breast Cancer Cells Exposed to Ascididemin

**DOI:** 10.3390/md11103846

**Published:** 2013-10-11

**Authors:** Daniel Morvan

**Affiliations:** 1 Faculty of Medicine, University of Auvergne-UDA, 28 Place Henri Dunant, BP 38, Clermont-Ferrand F-63001, France; E-Mail: Daniel.Morvan@udamail.fr; Tel.: +33-473-178-458; 2 Comprehensive Cancer Centre Jean Perrin, 58 rue Montalembert, Clermont-Ferrand F-63011, France

**Keywords:** ascididemin, breast adenocarcinoma cells, metabolomics, biomarkers, metabolic pathway discovery, oxidative stress, inflammation

## Abstract

Marine natural products are a source of promising agents for cancer treatment. However, there is a need to improve the evaluation of their mechanism of action in tumors. Metabolomics of the response to anti-tumor agents is a tool to reveal candidate biomarkers and metabolic targets. We used two-dimensional high-resolution magic angle spinning proton-NMR spectroscopy-based metabolomics to investigate the response of MCF7 breast cancer cells to ascididemin, a marine alkaloid and lead molecule for anti-cancer treatment. Ascididemin induced severe oxidative stress and apoptosis within 48 h of exposure. Thirty-three metabolites were quantified. Metabolic response involved downregulation of glycolysis and the tricarboxylic acid cycle, and phospholipid metabolism alterations. Candidate metabolic biomarkers of the response of breast cancer cells to ascididemin were proposed including citrate, gluconate, polyunsaturated fatty acids, glycerophospho-choline and -ethanolamine. In addition, candidate metabolic targets were identified. Overall, the response to Asc could be related to severe oxidative stress and anti-inflammatory effects.

## 1. Introduction

Marine natural products are a source of promising molecules for cancer treatment. However, there is a need to improve the evaluation of their mechanism of action in tumors. Metabolomics is one of the latest technologies for global and fast phenotyping of gene expression or response to drugs or nutrients, with the potential of metabolic biomarker or pathway discovery [1,2,3,4]. Well recognized tools for metabolomics are NMR spectroscopy which is limited in sensitivity but shows high metabolite specificity and remarkable versatility of acquisition conditions, and gas or liquid chromatography-mass spectrometry which exhibits much better sensitivity, but displays selectivity of analysis and requires systematic material extraction. Among NMR spectroscopy techniques, the high resolution magic angle spinning (HRMAS) technology is especially suited to acquisition in intact cells, thus avoiding extraction procedures, and issues of metabolite selectivity. Also, it provides metabolic phenotyping as close as possible to the real biochemical cell content. 

Quantification is necessary to ensure reproducibility of findings, proper biomarker identification, or steady state concentration-based data processing. We used 2D HRMAS proton NMR spectroscopy-based metabolite profiling [3,5] to get insights into the mechanisms of the response of MCF7 breast cancer cells to ascididemin (Asc), a marine alkaloid extracted from the Mediterranean ascidian *Cystodytes dellechiajei*. 

Asc acts as a topoisomerase II inhibitor, and exerts strong anti-proliferative effects in several tumor cell lines including MCF7 breast adenocarcinoma cells, through induction of caspase-dependent apoptosis [6,7]. By itself, this highly toxic alkaloid has no value as an anti-cancer agent but is considered as the lead of a family of anti-cancer drug candidates [8]. After exposure to Asc, mechanisms leading to cell death involve reactive oxygen species (ROS) release due to reduction of Asc iminoquinone moiety and DNA break formation [8], followed by oxidative stress-dependent apoptosis through JNK-dependent activation of caspase-2 [9].

We found that early (6–24 h) response of MCF7 breast cancer cells to Asc involved alterations of glycolysis, tricarboxylic acid (TCA) cycle, amino acid and phospholipid metabolism, and accumulation of gluconic acid (Gna). The latter could be explained by glucose dehydrogenation, probably as a means to respond to oxidative stress. Candidate metabolic biomarkers of the response of breast cancer cells to Asc were proposed including citrate, Gna, polyunsaturated fatty acids which levels increase, and glycerophospho-choline and -ethanolamine which levels decrease. In addition, from steady-state concentration-based data analysis, evidence was given of metabolic pathways being altered in response to Asc that can be related to oxidative stress and anti-inflammatory responses.

## 2. Results and Discussion

### 2.1. Global Response of MCF7 Cells to Asc

**Figure 1:**
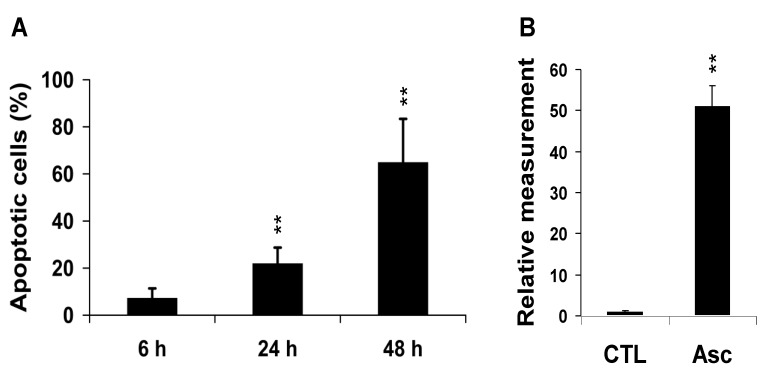
Global response of MCF7 breast cancer cells to Asc. (**A**) Percentage of apoptotic cells in attached cells after 6 h , 24 h, and 48 h treatment by Asc as determined by propidium iodide staining and FACS detection (*n =* 3 at each time). ** *P* < 0.01 Asc *vs.* CTL (untreated MCF7 cells); (**B**) Tail DNA quantification relative to the CTL value. ** *P* < 0.01, Asc (*n =* 3) *vs.* CTL (*n =* 3).

Treatment of MCF7 breast cancer cells with Asc yielded marked cell death at 48 h with 65% ± 5.8% (*P* < 0.01) apoptotic cells (Figure 1A). The count of apoptotic cells was 7% ± 4% (*P* = NS) and 22% ± 7% (*P* < 0.01) at 6 h and 24 h, respectively, indicating that critical metabolic events took place before 24 h. In Comet assays, tail DNA increased in MCF7 cells exposed to Asc at 24 h (×31, *P* < 0.01) (Figure 1B). 

### 2.2. NMR Spectrum Analysis of Asc-Treated MCF7 Cells

**Figure 2:**
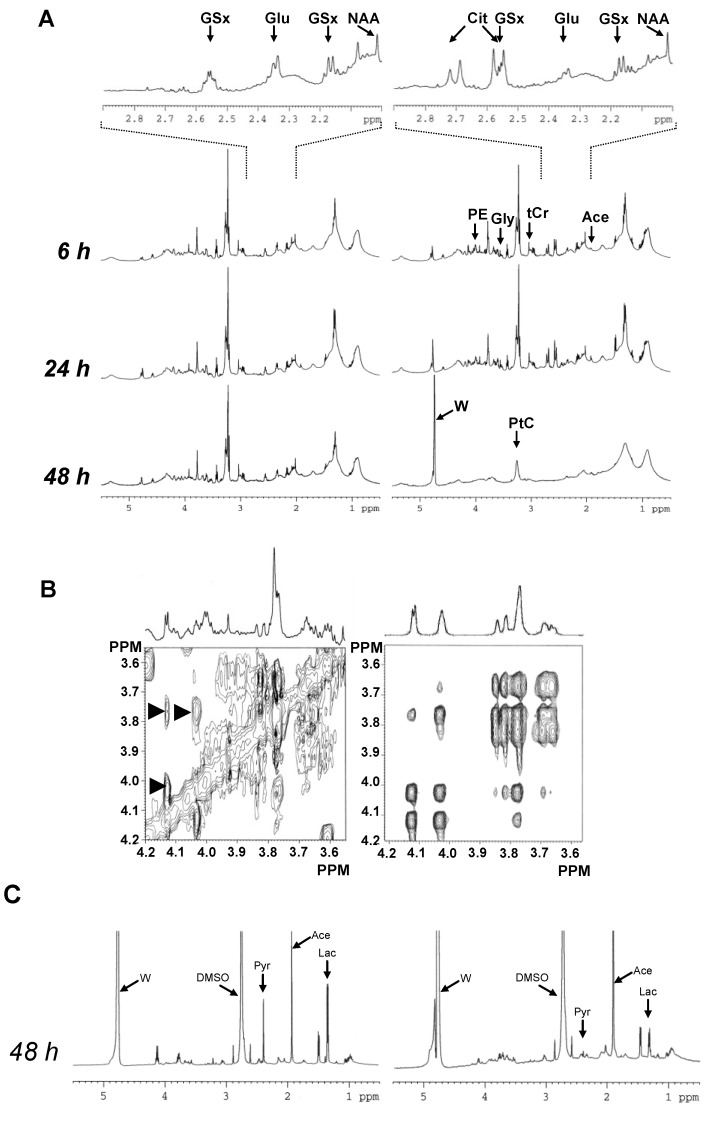
Global metabolic response of MCF7 breast cancer cells to Asc. (**A**) Typical 1D ^1^H-NMR spectra of untreated (left) and Asc-treated (right) intact MCF7 breast cancer cells at 6 h, 24 h and 48 h. Several metabolite signals are shown. For abbreviations, see Table 1. W, residual water signal; (**B**) Gluconic acid (Gna) NMR signals. Left, typical 2D TOCSY spectrum selection in the 3.55–4.20 ppm × 3.55–4.20 ppm area, with the corresponding 1D NMR spectrum above, in the Asc-treated group; Right, 2D TOCSY spectrum of Gna pure standard (www.bmrb.wisc.edu [10]). Arrowheads, correlation signals characterizing Gna; (**C**) Typical 1D ^1^H-NMR spectra of the culture medium of untreated (left) and Asc-treated (right) MCF7 breast cancer cells at 48 h. Several metabolite signals are shown. For abbreviations, see Table 1. Pyr, pyruvate; DMSO, dimethylsulfoxide.

A strong signal of Cit was visible in 1D NMR spectra at exactly 6 h after the onset of treatment. The Cit level increased between 6 h and 24 h. Other obvious metabolite variations involved increase of alanine (Ala, methyl signal at 1.47 ppm) and phosphoethanolamine (PE, methylene signal at 4.00 ppm). Between 24 h and 48 h, spectra of treated cells were completely upset, testifying the execution of apoptosis. At 48 h, most small metabolite signals had disappeared from spectra only leaving macromolecular and lipid signals, of which that of phosphatidylcholine (PtC) (Figure 2A). 

Analysis of the 3.50 × 4.20 ppm spectral domain of the Asc-treated group showed unusual signals at 4.12, 4.02, 3.83, 3.82, 3.76 and 3.66 ppm in 1D NMR spectra. Furthermore, 2D NMR spectra revealed strong correlations at 4.02 × 4.12 ppm, 3.76 × 4.12 ppm, 3.76 × 4.02 ppm, 3.66 × 3.76 ppm, and 3.83 × 3.66 ppm. A less intense correlation was observed at 3.66 × 4.02 ppm. The interrogation of the *BMRB* (www.bmrb.wisc.edu [10]) and *HMDB* (www.hmdb.ca [11]) spectral databases revealed that the unknown signals corresponded to gluconate (Gna) [12] (Figure 2B). 

To get some more information on the transition to apoptosis, the culture medium was analyzed using the same NMR sequences. At 48 h, it revealed, in the Asc-treated group, decreased lactate (Lac) level, providing evidence of downregulation of glycolysis, together with pyruvate consumption from the medium and increased acetate (Ace) release by the cells, testifying mitochondrial deficiency (Figure 2C). 

**Table 1:** Identified and quantified metabolites. Metabolites were arranged into five subsets: –: Glycolysis/TCA cycle/lipid metabolism derivatives (Glc, Lac, Ace, Cit, PUF, MyI, and Gna), –: Glutamate (Glu) derivatives (Glu, Gln, Pro, Ala, NAA, Asn, Asp, and Arg), –: Methionine (Met) and transsulfuration derivatives (Met, Ply, Hcy, tCr, GSx, hTa, and Tau), –: Other amino acids (Gly, Lys, Phe, and Thr), –: and Phospholipid metabolism derivatives (PE, GPE, Cho, PC, CDPC, PtC, and GPC). In the description of chemical shifts, a single chemical shift indicates a 1D-based measurement, and a combination of chemical shifts a 2D-based measurement. ×, correlation between two chemical shifts; &, correlations used for quantification.

Metabolite subset	Abbreviation	Metabolite	Chemical shifts (ppm) in 1D and 2D NMR spectra
Glycolysis/TCA cycle/lipid metabolism	Glc	β-glucose	3.25 × 4.65 & 3.49 × 4.65
	Lac	Lactate	1.34 × 4.11
	Ace	Acetate	1.92
	PUF	Polyunsaturated fatty acids	2.79 × 5.33
	Cit	Citrate	2.55 × 2.75
	MyI	Myoinositol	3.54 × 3.48 & 3.61 × 3.48
	Gna	Gluconate	3.83 × 4.12 & 4.02 × 4.12
Glutamate	Glu	Glutamate	2.06 × 3.76
	Gln	Glutamine	2.12 × 2.46
	Pro	Proline	2.03 × 4.15
	Ala	Alanine	1.47 × 3.77
	Asn	Asparagine	2.88 × 3.99 & 2.95 × 3.99
	Asp	Aspartate	2.70 × 3.89 & 2.95 × 3.89
	NAA	*N*-acetyl-aspartate	2.50 × 4.40 & 2.70 × 4.40
	Arg	Arginine	1.68 × 3.23 & 1.92 × 3.23
Methionine and transsulfuration	Met	Methionine	2.14 × 2.63 & 2.20 × 2.63
	Ply	Polyamines	1.80 × 3.10 & 2.13 × 3.10
	Hcy	Homocysteine	2.17 × 2.72
	tCr	Total creatine	3.03
	GSx	Total glutathione	2.17 × 2.55 & 2.55 × 4.56
	hTa	Hypotaurine	2.63 × 3.35
	Tau	Taurine	3.27 × 3.43
Other amino acids	Gly	Glycine	3.56
	Lys	Lysine	1.90 × 3.77
	Phe	Phenylalanine	3.13 × 3.99
	Thr	Threonine	1.32 × 3.58
Phospholipid metabolism	PE	Phosphoethanolamine	3.22 × 3.99
	GPE	Glycerophosphoethanolamine	3.30 × 4.12
	Cho	Choline	3.55 × 4.07
	PC	Phosphocholine	3.62 × 4.18
	CDPC	Cytidine diphosphate choline	3.66 × 4.42
	PtC	Phosphatidylcholine	3.26
	GPC	Glycerophosphocholine	3.66 × 4.34

**Figure 3:**
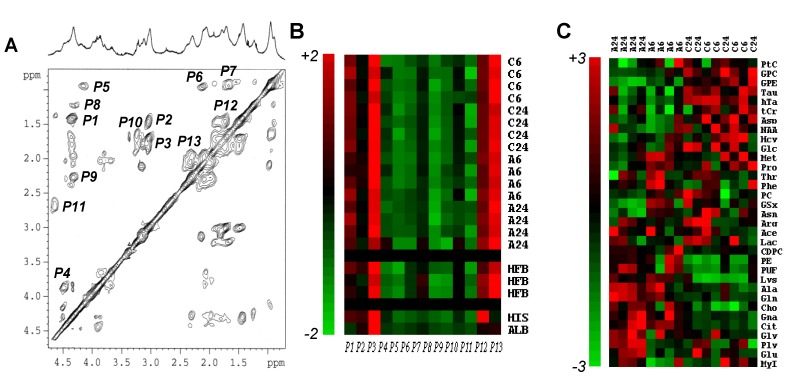
NMR-based proteomics and metabolomics of MCF7 breast cancer cell response to Asc. (**A**) Typical 2D TOCSY spectrum of a protein extract of MCF7 cells, with its corresponding 1D NMR spectrum above. The attributed signals were numbered from *P1* to *P13*, as follows: *P1*, alanyl β–α; *P2*, lysyl γ–ε; *P3*, lysyl δ–ε; *P4*, seryl β–α; *P5*, leucyl δ–α; *P6*, valyl γ–β and γ’–β; *P7*, leucyl δ–β and δ–γ; *P8*, threonyl γ–β and γ–α; *P9*, glutamyl γ–α; *P10*, arginyl β–δ and γ–δ; *P11*, aspartyl β–α; *P12*, lysyl γ–δ; *P13*, glutamyl β–γ; (**B**) Color display of CPV measurements from protein extracts. Signals *P1* to *P13* were measured in 2D NMR spectra MCF7 cell protein extracts in *n =* 8 untreated (C6 and C24 hold for untreated control at 6 h and 24 h, respectively) and *n =* 8 Asc-treated (A6 and A24 hold for Asc-treated at 6 h and 24 h, respectively) MCF7 cells. Each individual measurement set was autoscaled. For comparison are shown data from protein extracts of human fibroblasts (HFB, *n =* 3), and commercially available calf thymus histone type IIa (HIS) and bovine serum albumin (ALB). Red, variations above mean up to mean + 2 × SD; Green, variations below mean down to mean − 2 × SD; (**C**) Hierarchical clustering of metabolic data. Hierarchical clustering of the whole set of individuals (*n =* 8 untreated *vs.* *n =* 8 Asc-treated MCF7 cell pellets) and metabolites (*n =* 33) showing clear separation between groups, and quite well-defined metabolite variations. A6 and A24, Asc-treated MCF7 cell independent samples at 6 h and 24 h; C6 and C24, untreated MCF7 cell independent samples at 6 h and 24 h. For metabolite abbreviations, see Table 1. Red, metabolite variations above average up to +3 × SD; Green, metabolite variations below average down to −3 × SD.

### 2.3. Metabolomics of Asc-Treated MCF7 Cells

As a preliminary step of the metabolomics method, which requires protein NMR signal identification, 13 2D NMR signals of proteins were assigned in ^1^H-NMR spectra of protein extracts of MCF7 breast cancer cells, untreated or exposed to Asc (Figure 3A). They were quantified using their cross-peak volume (CPV), and autoscaled. Only a small, not statistically significant, difference (6% of average protein signal) was found between 2D NMR spectroscopy protein signals of the untreated and Asc-treated groups. For comparison, the same calculations were performed on 2D NMR spectra of human fibroblast protein extracts (*n =* 3) and commercially-available histones and albumin. Treatment with Asc did not altered protein content, which established the full applicability of the used metabolomics method [6]. Also, the protein composition of human fibroblasts varied lowly in comparison with that of MCF7 breast cancer cells. Only pure protein standards differed in amino-acid composition (Figure 3B). 

Identified and quantified metabolites were gathered into five metabolite subsets (Table 1): glycolysis/TCA cycle/lipid metabolism derivatives (Glc, Lac, Ace, PUF, Cit, MyI, Gna), Glu derivatives (Glu, Gln, Pro, Ala, Asn, Asp, NAA, Arg), Met and transsulfuration derivatives (Met, Ply Hcy, tCr, GSx, hTa, Tau), other amino acids (Gly, Lys, Phe, Thr), and phospholipid metabolism derivatives (PE, GPE, Cho, PC, CDPC, PtC, GPC). 

As an unsupervised multivariate statistical description of metabolite alterations induced by Asc, we used hierarchical clustering of the whole set of data (Figure 3C). The analysis discriminated the response to Asc. Metabolites accumulating in response to Asc were Cit, Gna, and phospholipid derivatives (Cho, CDPC, PE), while decreasing metabolites were other phospholipid derivatives (GPE, GPC, PtC) and transulfuration pathway derivatives (Hcy, hTa, Tau). 

**Figure 4:**
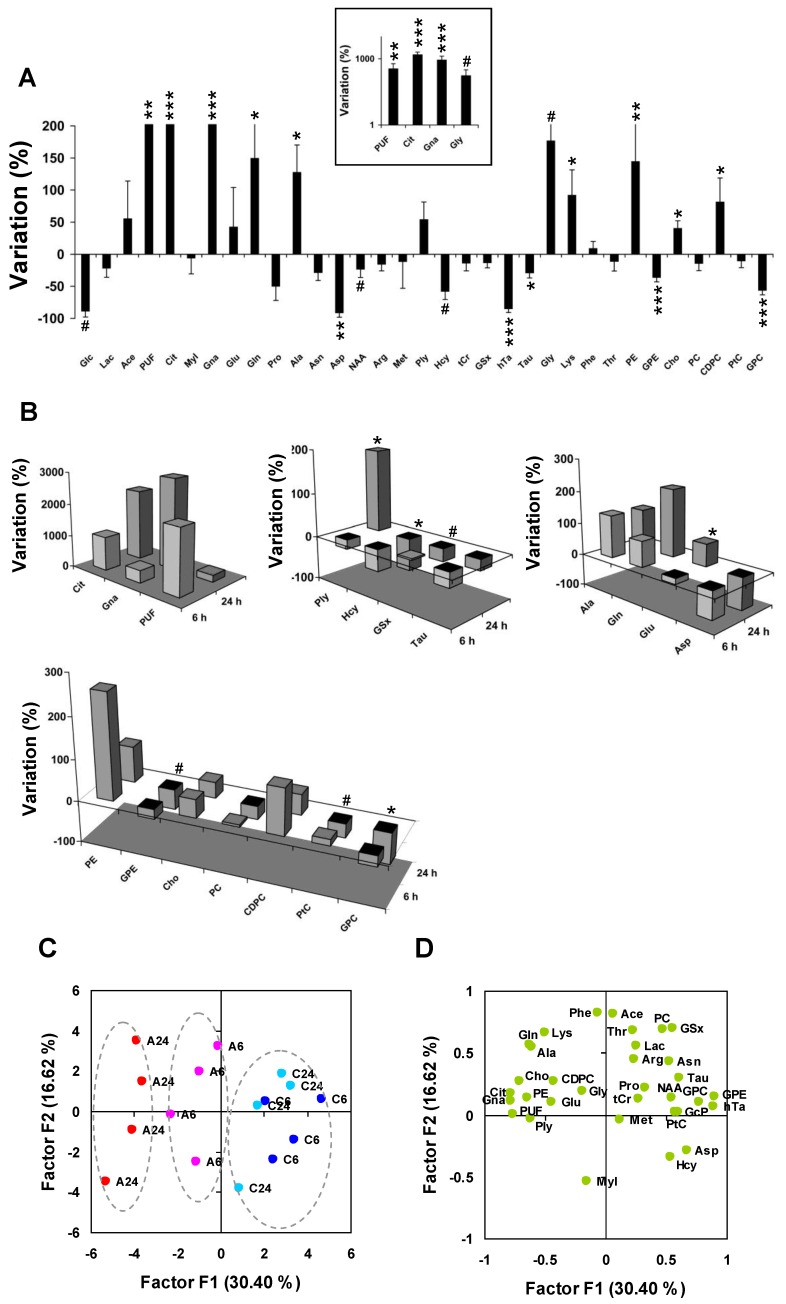
Differential metabolite profiling and principal component analysis. (**A**) Differential quantitative metabolite profiling of the early response to Asc (*n =* 8 Asc-treated *vs.* *n =* 8 untreated MCF7 cell pellets). Inset: log scale for the four metabolites with the largest variations. Abbreviations, see Table 1. * *P* < 0.05; ** *P* < 0.01; *** *P* < 0.001 (two-tailed Mann-Withney test); ^#^ *P* < 0.05 (one-tailed test); (**B**) Comparison between 6 h and 24 h shown as three-dimensional histogram plots using average variations between *n =* 4 Asc-treated and *n =* 4 untreated at 6 h and 24 h, respectively. From left to right, and top to bottom: Metabolites from the glycolysis/TCA cycle/lipid metabolism derivative subset, Met and transsulfuration derivatives, Glu derivatives, and phospholipid metabolism derivatives. At 6 h, most biochemical processes associated to oxidative stress and cellular defense were initiated. * *P* < 0.05; ^#^ *P* < 0.05 (one-tailed test); (**C**,**D**) Principal component analysis of metabolic data; (**C**) The individual plot shows that the main axis (F1) separates samples responding to Asc (left; 24 h, red; 6 h, purple), from control samples (right, 24 h, light blue; 6 h, dark blue); (**D**) The loading plot shows correlation of metabolites with the main axes. C6 and C24, untreated at 6 h and 24 h; A6 and A24, Asc-treated at 6 h and 24 h. For metabolite abbreviations, see Table 1.

Then, we calculated average metabolite variations in response to Asc. The largest three changes corresponded to the accumulation of PUF (×4.6, *P* < 0.01, *n =* 8 untreated *vs. n =* 8 Asc-treated cell pellets), Cit (×16.8, *P* < 0.001), and Gna (×10.2, *P* < 0.001) (Figure 4A). Glycolysis alterations were observed with decrease of Glc (−75% ± 22%, *P* < 0.05, one-tailed test), and moderate decrease of Lac (−21% ± 14%). Significant alterations occurred in the Glu derivative group with increase of Gln (+150% ± 56%, *P* < 0.05) and Ala (+128% ± 42%, *P* < 0.05), and severe decrease of Asp (−92% ± 7%, *P* < 0.01). The Met derivative subset was strongly altered with decrease of Hcy (−58% ± 12%, *P* < 0.05, one-tailed test), hTa (−85% ± 6%, *P* < 0.001), and Tau (−29% ± 8%, *P* < 0.05), and moderate decrease of GSx (−13% ± 8%). The phospholipid metabolism subset was strongly modified with increase of PE (+145% ± 59%, *P* < 0.01), Cho (+41% ± 11%, *P* < 0.01) and CDPC (+82% ± 37%, *P* < 0.05), decrease of GPE (−36% ± 6%, *P* < 0.001) and GPC (−57% ± 7%, *P* < 0.001), and mild variation of PtC (−11% ± 10%).

Despite the limited number of samples at 6 h and 24 h, we sought for time-varying response to Asc. Cit and Gna increased between 6 and 24 h, and PUF decreased, but these variations did not reach statistical significance (Figure 4B). However, significant variations were observed for other metabolites between 6 and 24 h: Ply (+211%, *P* < 0.05, *n =* 4 at 6 h *vs.* *n =* 4 at 24 h, in the Asc-treated group), Met (−132%, *P* < 0.05, one-tailed test), Glu (+87%, *P* < 0.05), GSx (−32%, *P* < 0.05, one-tailed test), Hcy (−25%, *P* < 0.01), Pro (−87%, *P* < 0.01), Asn (−22%, *P* < 0.05), GPE (−25%, *P* < 0.05, one-tailed test), PtC (−48%, *P* < 0.05, one-tailed test), and GPC (−49%, *P* < 0.05). 

Principal component analysis was used to condense information carried by data. The first axis (F1), accounting for 30% of total information and separating Asc from control samples, opposed the set of Gna, Cit, and PUF to the set of GPE, hTa, and GPC, all candidate biomarkers of the response to Asc (Figure 4C,D). High levels of Gna, Cit and PUF and low levels of GPE, hTa and GPC characterized Asc-treated cells, even more after 24 h than 6 h treatment. There was a trend for an opposing variation between Glu and Asp along the F1 axis. The second axis (F2) accounted for 17% of total information, and was explained by opposed variations of MyI and Hcy on one side, and Ace, PC and GSx on other side.

**Figure 5:**
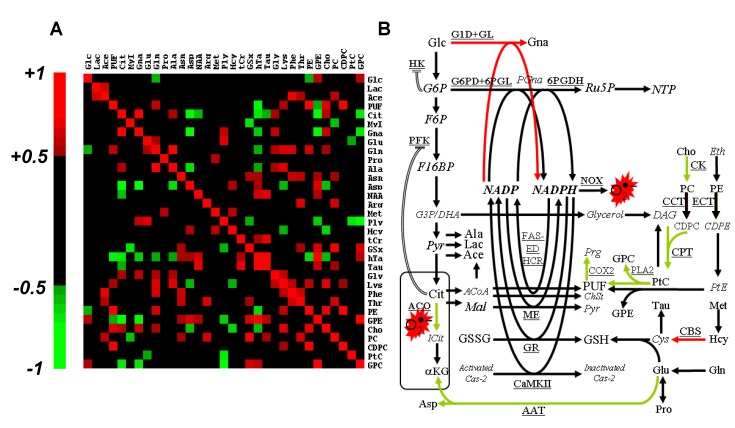
Steady-state concentration-based data processing and interpretation. (**A**) Spearman’s rank total correlation coefficient between metabolites. Statistical significance (*P* < 0.05) is obtained for |r| > 0.50, and only these coefficients are color-coded; (**B**) Interpretative scheme of the response to Asc. *Italicized*, non-detected metabolites; red arrows, activated pathways; green arrows, inhibited pathways; double lines, allosteric regulation. G6P, glucose-6-phosphate; PGna, 6-phospho-gluconate; Ru5P, ribulose-5-phosphate; NTP, nucleosides tri-phosphate; F6P, fructose-6-phosphate; F16BP, fructose-1,6-bisphosphate; G3P, glyceraldehyde-3-phosphate; DHA, dihydroxyacetone; Pyr, pyruvate; DAG, diacylglycerol; Eth, ethanolamine; CDPE, cytidine diphosphate ethanolamine; PtE, phosphatidylethanolamine; Prg, prostaglandin; ICit, iso-citrate; αKG, α-keto-glutarate; ACoA, acetyl-CoA; Mal, malate; ChSt, cholesterol; Cys, cystein; Cas-2, caspase-2; G1D, NAD(P)H-dependent glucose-1-dehydrogenase; GL, gluconolactonase; G6PD, glucose-6-phosphate dehydrogenase; 6PGDH, 6-phosphogluconate-dehydrogenase; NOX, NADPH oxidase; FAS-ED, fatty acid synthase, fatty acid elongase and desaturase; HCR, 3-hydroxy-3-methylglutaryl-CoA reductase; CCT, CTP: phosphocholine cytidylyltransferase; ECT, CTP: phosphoethanolamine cytidylyltransferase; COX2, cyclo-oxygenase-2; ME, malic enzyme; GR, glutathione reductase; GS, glutathione synthase; CBS, cystathionine-β-synthase; CaMKII, NADPH-dependent calcium/calmodulin-dependent protein kinase II; AAT, aspartate aminotransferase. Other abbreviations, see Table 1.

The rank correlation matrix was calculated to highlight short- and long-range co-variations between metabolites of slow turn-over or blocked at rate-limiting enzymes (Figure 5A). Correlations that implicated Gna, Cit and PUF—the three biomarkers of oxidative stress—revealed long-range metabolic relationships. Unsurprisingly, Gna strongly correlated with Cit (*r* = +0.88, *P* < 0.01). Actually, Cit is an allosteric inhibitor of phosphofructokinase (PFK), a well-known regulatory process used by the cell to enhance NADPH production. Gna, the witness of a novel source for NAD(P)H production, correlated negatively with transsulfuration derivatives of which GSx, the main cellular NADPH consumer. GPC and GPE correlated negatively with Cit and Gna, and positively with metabolites with an antioxidant role (hTa, GSx).

Taken together, metabolite variations testified severe alteration of glycolysis, TCA cycle, Glu and phospholipid metabolism, and redox-sensitive transsulfuration. Changes in Hcy and GSx were suggestive of the enrolment of transsulfuration in glutathione biosynthesis, and upregulation of cystathionine-β-synthase (CBS) in Asc-treated cells. Opposing variations in Glu and Asp may suggest downregulation of aspartate aminotransferase (AAT). PUF accumulation may be related to downregulation of cyclooxygenase-2 (COX-2), which is a component of anti-inflammatory response. Phospholipase A2 (PLA2) activity has been shown to correlate with GPC and GPE release [13], and the decrease of GPC and GPE, in this study, could be the consequence of anti-inflammatory response which downregulates COX-2 and PLA2 [14]. 

Cho and CDPC accumulation suggests inhibition of choline-kinase and inhibition of CDP-choline:1,2-diacylglycerol cholinephosphotransferase (CPT). Accumulation of Cit indicates blockade of mitochondrial/cytosolic aconitase in response to Asc, most likely through ROS oxidation of [4Fe-4S]^2+^ center [15]. This mechanism would explain Cit diffusion out of mitochondria, and accumulation into the cytosol. Cytosolic Cit is a well-known allosteric inhibitor of PFK [16], thus downregulating glycolysis, and upregulating NADPH-producing pathways. Alternatively, Cit may become a source of NADPH through conversion into malate and activity of malic enzyme.

Gna accumulation could result from either glucose-6-phosphate dehydrogenase (G6PD) rate-limitation or hexokinase (HK) blockade. Among glucose-6-phosphate-regulated hexokinases, HK-II is mostly bound to the mitochondrial membrane, and its inhibition detaches it from the mitochondrial membrane, yielding loss of mitochondrial potential and apoptosis [17]. Gna was reported to be increased in fibroblast lines transfected with cancer-causing genes [18]. However, it is the first time that Gna is reported to be implicated in human tumor cell response to a pro-oxidant agent.

A summary of the response to severe oxidative stress as derived from the present metabolomics study is given in Figure 5B. It is centered on the balance between NADPH production and consumption, and depicts the metabolic counterpart of ROS production and scavenging. Although the molecular chemistry of ROS deserves extensive work, little is known about central metabolism alterations associated to severe oxidative stress. It was recently shown that ROS production could be evaluated through the activity of the pentose phosphate pathway [19]. However, to the best of our knowledge, little or no research has reported the metabolic counterpart of ROS production and scavenging.

## 3. Experimental Section

### 3.1. Chemicals and Reagents

D_2_O (SDS) was the NMR solvent and locking medium. Histone type IIa from calf thymus, and bovine serum albumin (Sigma Aldrich), were used for NMR spectroscopy assignments and method development. Ascididemin (Asc), 9*H*-quino[4,3,2-de][1,10]phenanthroline-9-one, a natural marine alkaloid extracted from the Mediterranean ascidian *Cystodytes dellechiajei*, was a gift from Bernard Banaigs (Laboratoire Arago, Université de Perpignan, France). It was solubilized in dimethylsulfoxide (DMSO, Merckeurolab) immediately before use in the culture medium, where the DMSO concentration was maintained at a final concentration of 0.5%. Eagle’s MEM-Glutamax medium, solution of vitamins, sodium pyruvate, non-essential amino acids and phosphate buffered saline solution (PBS), and gentamicin base were purchased from Gibco-BRL. Fetal calf serum was sourced from Bio West (Nuaillé, France). Propidium iodide was purchased from Molecular Probes (Invitrogen, Cergy-Pontoise, France). 

### 3.2. Cell Culture

Human estrogen-responsive breast adenocarcinoma MCF7 cells were purchased from the European Collection of Cell Cultures (ECACC), and cultured at 37 °C under 5% CO_2_ in Eagle’s MEM-Glutamax medium supplemented with 10% (vol/vol) fetal calf serum, vitamin, sodium pyruvate, non-essential amino acids, and gentamicine base. Normal human dermal fibroblasts were purchased from Promocells. Cells were maintained in exponential growth at 37 °C in humidified atmosphere containing 5% CO_2_, using the same medium and supplementations than MCF7 cells. MCF7 cells were treated with the solvent (DMSO 0.5%) containing Asc (5 µM) or the solvent alone (negative control). At specified times (6, 24, and 48 h after the onset of treatment), cells were harvested by trypsinization, rinsed once with PBS then 2 times with PBS-D_2_O (96 mg PBS in 10 mL D_2_O). Cell pellets were then stored at −80 °C until exploitation.

### 3.3. Detection and Quantification of Apoptosis

Apoptosis was determined by (i) microscopical analysis of cell morphology with fluorescence microscope (λ = 365 nm; *G* × 600) after cell staining with Hoechst 33342 (0.5 µg/mL, 10 min in the dark), and (ii) by fluorescence-activated cell sorting (FACS) with a FACScalibur (Becton Dickinson) after cell staining with propidium iodide. The percentage of apoptotic cells was quantified as the amount of cells in sub-G1 phase.

### 3.4. Comet Assay

The measured Tail DNA value, corresponding to the amount of DNA in the comet tail, increased proportionally with the number of DNA strand breaks induced by oxidative stress. DNA strand breaks in MCF7 cells were quantified using the alkaline version of the Comet assay adapted from [20].

### 3.5. Protein Extraction

All the steps were done in an ice-cold environment. An amount of ~10^7^ cells was suspended in 2 mL methanol/chloroform (2:1, vol/vol) and ultrasonicated for 1 min. 500 µL of water/chloroform (1:1, vol/vol) were added and the two phases (organic and aqueous) were separated by centrifugation (1500× *g*, 20 min, 4 °C). The inter-phase precipitate and the aqueous phase were collected. To optimize protein extraction and purification, the process was repeated 2 times. Cellular extract pH was adjusted to 7.20. The protein precipitates and aqueous phases were evaporated under argon flux, lyophilized, and stored at −80 °C until further use. Before use, each freeze-dried protein or aqueous phase extract was reconstituted in PBS–D_2_O. The internal chemical shift reference was 3-(trimethylsilyl)-1-propanesulfonic acid sodium salt (TPS, 1% in D_2_O solution).

### 3.6. NMR Spectroscopy Analysis

NMR Spectroscopy was performed on a small bore 500 MHz Avance DRX spectrometer (Bruker Biospin, Karlsruhe, Germany) equipped with a high resolution magic angle spinning (HRMAS) probe. Unprocessed cell pellets, water-soluble extracts, or protein extracts were set into 4 mm diameter 50 µL free volume zirconium oxide rotor tubes. Rotors were spun at 4 kHz, and cooled at 4 °C using the BCU-05 temperature unit.

One-dimensional (1D) proton NMR spectra were obtained using a Nuclear Overhauser Enhancement spectroscopy sequence with low power water signal presaturation (NOESYPR) during both the 3.8-s relaxation delay and the 100-ms mixing time of the sequence. The spectral width was 12 ppm with 16 K complex data points and 32 transients. This resulted in 2:50 min acquisition duration. After Fourier transformation, a baseline correction was applied using a spline function. One-dimensional NMR spectra were processed using deconvolution procedures (TOPSPIN 1.3 software, Bruker Biospin).

After the 1D acquisition, a two-dimensional (2D) NMR spectrum was immediately recorded using a Total Correlation Spectroscopy (TOCSY) sequence involving water signal suppression at low power, 6-ppm spectral bandwidth along both frequency axes, 256 samples along the first axis, and 2000 samples along the second axis, 75-ms mixing time during which was applied the spin-lock pulse train (DIPSI-2), 1-s relaxation delay, and 16 repetitions. The 2D NMR spectrum duration was 1:41 h. TOCSY spectra were first reconstructed both at high resolution (2000 × 256), and referenced to TPS for assignments. Then, they were reconstructed at lower resolution (256 × 256) for quantification. Baseline correction was applied using a second order polynomial. The latter spectra were transferred on an EXCEL worksheet (Microsoft Co.) and processed using a homebuilt routine designed to automatically compute spectral cross-peak volumes (CPVs) according to the developed quantitative method. 

Metabolites cross-peak signals normalized to protein CPVs, thus provided a metabolite measurement proportional to a concentration, according to the procedure of reference [6].

### 3.7. Statistical Analysis

Comparison between treated and control metabolite measurements was done using the nonparametric Mann-Whitney test. The full set of data (table of *n =* 8 independent control and *n =* 8 independent treated samples, and *n =* 33 metabolites) was processed using multivariate statistical analysis. Briefly, hierarchical clustering aims at identifying, among all individuals (samples) and metabolites, clusters of individuals and metabolites who behave similarly. Principal component analysis aims at condensing information contained in data. It provides a small number of factors (most often 2), new variables and linear combinations of metabolites that explain the largest part of information contained by data (the percentage of explained information is given with each factor). It highlights the most informative metabolic trends within the whole set of data. The strength of the correlation of individual metabolites with factors is the assigning of a metabolic/biochemical meaning to the factors, and is given by the loading plot. Individual samples projected onto the factors (individual plot) may clusterize into clouds with functional meaning. Hierarchical clustering of data used the rank correlation (Spearman coefficient) as the distance criterion, and mean linkage as the clustering criterion Principal component analysis used the rank correlation of data (as a preferred alternative to the Pearson correlation, given the non-gaussian structure of data), Correlation between metabolites used the Spearman’s nonparametric rank correlation. Statistical tests were two-tailed unless specified. Univariate and multivariate statistical analyses were performed using the Xlstat 7.5 software (Addinsoft).

## 4. Conclusions

Two-dimensional HRMAS proton NMR spectrum-based metabolomics was successfully applied to evaluate the response of MCF7 breast cancer cells to Asc. Candidate metabolic biomarkers of the response to Asc involved citrate, gluconate and polyunsaturated fatty acids which levels increase, and glycerophospho-choline and -ethanolamine which levels decrease. In addition, evidence was given of candidate metabolic targets including aconitase and glycolysis. Finally, central metabolism alterations after exposure of breast cancer cells to Asc were shown to depict the metabolic counterpart of severe oxidative stress and anti-inflammatory responses.
